# The role of dogs is associated with owner management practices and characteristics, but not with perceived canine behaviour problems

**DOI:** 10.1038/s41598-024-77400-y

**Published:** 2024-11-12

**Authors:** Laura Gillet, Barbara Simon, Eniko Kubinyi

**Affiliations:** 1 Department of Ethology, Eötvös Loránd University, Budapest, Hungary; 2 MTA-ELTE Lendület “Momentum” Companion Animal Research Group, Budapest, Hungary; 3 ELTE NAP Canine Brain Research Group, Budapest, Hungary

**Keywords:** Human behaviour, Animal behaviour

## Abstract

Owned dogs are often regarded as family members, best friends, or even as substitutes for children. These new roles and their effects on human well-being have received scientific attention, but little is known about how attributing such social functions to dogs might influence the caring behaviour of the owner, which in turn can impact canine welfare. Our online survey of Hungarian dog owners (N = 790) aimed to describe what owners appreciate the most and the least in their dogs and to investigate the relationship between the social and non-social functions of the dog, the characteristics of the owner and the dog, the management practices of the owner, as well as the dog’s behaviour, including behaviour problems. We found that most owners appreciated petting and physical contact with their dog (97.6%), followed by ‘unconditional love’ (93.7%), the dog’s beauty (88.4%), and walking with the dog (86.5%). The most frequently mentioned problems were socialisation issues (20.4%) and lack of time (15.9%). Regarding canine behaviour, jumping up (33.2%), chasing animals (28.5%), territoriality (26.7%), and overexcitement (20.9%) were common problems. Almost a quarter of owners rated their dogs as totally obedient and 30.6% as totally safe off-leash. We identified three profiles of dog owners based on the roles they attribute to their dogs. Dogs with both companionship and practical functions (31.1%) were perceived as more obedient and provided higher safety benefits to their owners than others. Dogs regarded as ‘companion animals’ (19.4%) spent less time with their owners and had older owners than others. Lastly, owners labelled as ‘dog parents’ (49.5%) were more likely to keep their dog indoors only than others. Importantly, the roles attributed to dogs were not associated with owner-reported canine behaviour problems. We concluded that while the functions humans attribute to dogs are multiple and appear to have direct implications for the dogs’ daily lives, these variations in management practices do not necessarily seem to compromise their welfare.

**Supplementary Information:**

The online version contains supplementary material available at 10.1038/s41598-024-77400-y.

## Introduction

Since their domestication, dogs have occupied various utilitarian roles in human lives, such as hunting, herding, guarding, and fighting in wars^1,2^. This approach to dogs as useful animals can be described as a dominionistic, hierarchical orientation towards dogs, positioning humans above them^3^. However, humanistic orientations now seem to have become predominant in Western countries. Several socio-cultural factors, such as the spread of dog ownership among the middle class of the population during the Industrial Revolution, may explain this shift in attitudes towards dogs^3,4^. Hereafter, the term ‘dogs’ will refer specifically to dogs owned by one or more people and living close to human families. The large majority of people who share their lives with a dog consider their dog an integral family member^5–7^. Dogs have also been described as humans’ best friends, or even as child substitutes^3,8,9^, providing an avenue for childless owners to develop their parenting skills or for older couples to fill the void left by departed adult children^10^. Some owners (12–66% in representative and convenience Hungarian samples, respectively) even consider their dog to be the most important individual in their lives, even if they have children^6,11^. Family member, friend or child, these various roles embody the social functions attributed to a growing number of animals living in human families, as expounded by Veevers^12^ and Doré et al.^13^.

The noticeable growth of social roles in dogs may be paralleled by the demographic shift observed in post-industrial societies. Many factors, including falling fertility rates (i.e., number of births per woman) and increasing urbanisation, have led to the reorganisation of traditional human social networks^14^. Kinship networks are particularly affected, as the number of relatives within a family is impacted by the number of children per woman. This important change in the environment might explain, at least partly, the growing trend of domesticated animals playing social roles in their owners’ lives, as social support provided by kin has become less accessible^4^. It is especially notable in the case of dog keeping, as dogs are assumed to possess outstanding ‘human-like’ social skills^15–17^, which may enhance their adaptability to human social environments in comparison to other species, such as cats^18^.

Therefore, demographic factors like age, marital status, household composition, and parenthood, might play important parts in the perception of one’s dog’s role. Results reported by Pirrone et al.^19^ suggest that divorced, childless owners are more likely to consider their dog a child. Likewise, Albert and Bulcroft^5^ found high levels of anthropomorphism (i.e., the attribution of human characteristics, emotional and mental states and behaviours to non-human agents) and a stronger pet attachment among people without a romantic partner and childless couples. Although this last study did not specifically focus on the perceived role of the pet, Bouma et al.^18^ have suggested that pets’ social roles (e.g., child, friend, family member) could mediate the relationship between anthropomorphism and perceived social support from pets, as the attribution of human-like mental capacities to animals may be a prerequisite for their perception as social agents, and thus for the formation of affective bonds with them^20^.

Interestingly, the effects of these new roles of domesticated animals, particularly dogs, on human well-being have received more attention than their consequences for animal welfare (e.g.,^21,22^). Indeed, studies focusing on how assigning human-like roles to dogs influences owners’ caring behaviour are still rare. While some authors have described positive outcomes of anthropomorphic perceptions on pro-animal attitudes^23,24^ and on the dog-owner relationship quality^25^, others have warned of its possible harmful consequences for canine welfare due to inadequate care behaviours^20,26–28^. In the present article, we chose to focus on the owner’s management practices, as they can impact the daily well-being of dogs. We also considered canine behaviour problems as a welfare indicator, since they may result from inadequate management practices.

Canine behaviour problems are well documented, and their prevalence in the dog population can be quite high (e.g.,^29–31^). Risk factors for canine behaviour problems have also been extensively explored. However, the role of the dog tends to be missing in these studies; and when it is included, it is often dichotomized (companion dog versus working dog). Additionally, studies on the effects of the role attributed to the dog by its owner on the dog’s behaviour report variable results^28,32^. For example, Kubinyi et al.^33^ found that dogs kept exclusively as family members were less calm, less trainable, but bolder than dogs kept as family members in addition to other purposes (i.e., hobby, guarding, working, breeding), while Pirrone et al.^19^ reported that owners who considered their dog a child were more likely to complain about the dog’s attention-seeking behaviour. In contrast, Voith et al.^34^ did not show any relationship between the anthropomorphic attitudes of the owner and problematic canine behaviours.

The role assigned to the dog can also have a direct influence on its daily life. Chira and colleagues^35^ found that, in a global sample, the number of functions filled by dogs was positively correlated with a positive treatment and a ‘humanised’ perception of dogs (e.g., dogs were more likely to be treated as ‘close friends’, to be named or talked to). Meyer et al.^26^ highlighted the various challenges often encountered by dogs kept as companions, including high dependency on their owner, social isolation from conspecifics and unrealistic demands in terms of social interaction with humans. Ideal companion dogs are expected to be friendly, affectionate, and to enjoy being petted^36^. Yet, some dogs do not tolerate close physical interactions (e.g., hugs, kisses), although it happens frequently in daily owner-dog interactions. Dogs might display signs of distress that can be misread or overlooked by the owners, sometimes leading to negative outcomes such as biting^26,28^.

On a more practical level, the dog’s role might also be related to specific management and care practices. Owners describing their dogs as children often invest a lot into their dogs’ care and integrate them into family activities (e.g., walks and visits to dog parks, play, grooming)^37^. Marinelli et al.^38^ found that dogs acquired for companionship reasons were more likely to receive veterinary assistance than dogs acquired for work purposes or for ‘no reason’. Likewise, owned dogs with a companion role were found to be more often confined to their owners’ properties (i.e., not allowed to leave the owner’s property and roam in public and natural areas without supervision) than working dogs^39^. Additionally, dogs that have access to their owner’s bathroom, bedroom and bed might have acquired a status of a close, intimate family member^40^, as opposed to dogs kept as ‘pets’ only, that are not always allowed free access to their owners’ house^37^.

### The present study

Since studies investigating the interconnection between the perception of the dog-owner relationship and its consequences on the daily life of dogs are still scarce, our aim is to determine how roles that owners attribute to their dogs are associated with owner and dog characteristics, dog behaviour, as well as with management practices and perceived benefits derived from dog ownership. The latter were included because they may reflect the reasons why owners acquired their dogs, and what they expect from the relationship.

Regarding the perceived roles of the dog, we chose to include various social and non-social roles in our questionnaire, allowing dog owners to consider how well each role fits their dog. This also gave owners the opportunity to assign multiple roles to their dog, as dogs can be kept for more than one function^13,35^. This approach is relatively novel, as previous studies mostly employed single-choice questions when asking about the dog’s role. Yet, focusing solely on the main role of the dog can obscure the complexity of this aspect of the dog-owner relationship.

Based on previous findings, we predict that most dog owners will attribute a ‘family member’ role to their dogs. However, given that our scale offered the possibility to choose multiple roles, we expect to find specific patterns among dog owners varying in the predominant roles they attribute to their dogs. If that is the case, we hypothesize that owners attributing social roles to their dog, especially in terms of close, intimate relationships (i.e., *child*, *more important than any human*), will display specific characteristics, keep dogs with different characteristics and behaviours, and manage their dogs differently compared to owners who attribute both social and non-social roles (i.e., *assistance/guard-protection dog*, *domesticated animal*) to their dogs.

Identifying which dog and owner characteristics are associated with the social and non-social roles of dogs could help prevent inadequate care behaviours. For instance, if our results show that dogs with human-like roles are more fearful and anxious than dogs with animal-like roles, specific campaigns could educate owners on the particular needs of the dog species and how they differ from these of humans, while recognising and accounting for the deep emotional connection between the owner and their dog.

## Methods

### Ethics statement

The survey we used did not collect any sensitive or personal information about the owner. Participation in the study was voluntary and anonymous, and the owners were informed of the purpose of the study. The informed consent of all the participants was obtained at the beginning of the online questionnaire. Ethical approval was granted by the Hungarian United Ethical Review Committee for Research in Psychology; the ethical permission number is EPKEB 2022-62. All methods were carried out according to relevant guidelines and regulations for human participants.

### Subjects

We advertised our questionnaire through Facebook in our Hungarian Dog Ethology (‘Kutyaetológia’) group, which has more than 20,000 followers and our Hungarian Family Dog Project (‘Családi Kutya Program’) page with 7,400 followers. Data were collected between February and March 2022. In total, 799 participants answered the online questionnaire. Six participants were excluded because they rated each role of their dog the same number (5, 2 or 1), and three more were excluded after running the first cluster analysis for presenting unusual patterns of responses. Our sample is a convenience sample, over-representing women (87.1% vs. 56.3% in the Hungarian dog owning general population^41^) and under-representing older people (4.7% of our participants are aged 60 years or over vs. 23.3% in the Hungarian dog owning general population^41^). The characteristics of the respondents are summarised in Table 1.

**Table 1:** Descriptive results from questions regarding owners and their dogs (*N* = 790), with percentage distribution across answer categories.

**Variable and associated question**		**Categorical variable labels**			***N***			**%**
*1) Characteristics of the owner*								
**Gender**		Women			700			88.6
		Men			90			11.4
**Age group**		18–30 years			208			26.3
		30–40 years			210			26.6
		40–50 years			234			29.6
		More than 50 years			138			17.5
**Parental status**: do you have children?		Yes			280			34.8
		No			519			65.2
*2) Characteristics of the dog*								
**Sex**		Intact female			98			12.4
		Intact male			120			15.2
		Neutered female			326			41.3
		Neutered male			246			31.1
**Purebred status**		Purebred			509			64.4
		Mixed breed			281			35.6
**Age of the dog (in years)**		Mean age ± S.D: 4.99 ± 3.55						
**Place of acquisition**: where did you buy/adopt your dog?		Breeder (registered and non-registered breeder, puppy mill)			337			42.7
		Rescued dog (shelter, found on the streets, dog breed rescuers)			260			32.9
		Other (from family/acquaintance, own breeding/born at my place, other)			193			24.4
**Behaviour problems of the dog**: how typical are the following behaviour problems for your dog?		**Not typical at all (N**,** %)**	**Not typical (N**,** %)**	**I don’t know/I can’t decide/Neutral (N**,** %)**	**Typical (N**,** %)**		**Totally typical (N**,** %)**	
	House training problems	694 (87.8)	54 (6.8)	21 (2.7)	13 (1.6)		8 (1)	
	Escaping	654 (82.8)	69 (8.7)	31 (3.9)	28 (3.5)		8 (1)	
	Compulsive behaviour (e.g. circling)	642 (81.3)	80 (10.1)	32 (4.1)	30 (3.8)		6 (0.8)	
	Aggression to people	565 (71.5)	150 (19)	43 (5.4)	25 (3.2)		7 (0.9)	
	Too much whining	537 (68)	159 (20.1)	45 (5.7)	39 (4.9)		10 (1.3)	
	Chewing/Destruction	538 (68.1)	145 (18.4)	50 (6.3)	46 (5.8)		11 (1.4)	
	Food/toy protection	520 (65.8)	125 (15.8)	63 (8)	68 (8.6)		14 (1.8)	
	Rough play/Pinching	477 (60.4)	147 (18.6)	68 (8.6)	90 (11.4)		8 (1)	
	Separation anxiety	483 (61.1)	132 (16.7)	75 (9.5)	73 (9.2)		27 (3.4)	
	Faeces eating/Rolling in faeces	472 (59.7)	120 (15.2)	64 (8.1)	105 (13.3)		29 (3.7)	
	Fear of other people	416 (52.7)	190 (24.1)	64 (8.1)	102 (12.9)		18 (2.3)	
	Too much barking	406 (51.4)	181 (22.9)	92 (11.6)	81 (10.3)		30 (3.8)	
	Hard (or impossible) to call back during walks	363 (45.9)	217 (27.5)	108 (13.7)	78 (9.9)		24 (3)	
	Fear of other dogs	338 (42.8)	231 (29.2)	102 (12.9)	106 (13.4)		13 (1.6)	
	Noise phobia	395 (50)	167 (21.1)	80 (10.1)	107 (13.5)		41 (5.2)	
	Aggression to other dogs	322 (40.8)	234 (29.6)	100 (12.7)	113 (14.3)		21 (2.7)	
	Overexcitement	351 (44.4)	161 (20.4)	113 (14.3)	111 (14.1)		54 (6.8)	
	Fear of new things/situations	261 (33)	257 (32.5)	119 (15.1)	132 (16.7)		21 (2.7)	
	Territorial behaviour	390 (49.4)	106 (13.4)	83 (10.5)	148 (18.7)		63 (8)	
	Chasing wild/domesticated animals	296 (37.5)	155 (19.6)	114 (14.4)	151 (19.1)		74 (9.4)	
	Jumping up	265 (33.5)	160 (20.3)	103 (13)	196 (24.8)		66 (8.4)	
**Perceived obedience of the dog**: how obedient do you think your dog is?					**N**			**%**
		1 - Not totally obedient			175			22.2
		2			425			53.8
		3 - Totally obedient			190			24.1
**Off-leash safety**: how safe do you feel taking the leash off your dog on the street/public space/forest?		1 - Not totally safe			307			38.9
		2			241			30.5
		3 - Totally safe			242			30.6
*3) Management practices*								
**Tools and methods used to educate the dog**: what tools/methods do you use to train your dog?		**Never (N**,** %)**	**I have used it (N**,** %)**	**Often (N**,** %)**				
	Electric collar	739 (93.5)	41 (5.2)	10 (1.3)				
	Spiked collar	717 (90.8)	58 (7.3)	15 (1.9)				
	Muzzle	641 (81.1)	130 (16.5)	19 (2.4)				
	Training hygienic pad	612 (77.5)	153 (19.4)	25 (3.2)				
	Isolation	526 (66.6)	247 (31.3)	17 (2.2)				
	Physical discipline	494 (62.5)	285 (36.1)	11 (1.4)				
	Cage/Room kennel	491 (62.2)	192 (24.3)	107 (13.5)				
	Clicker	335 (42.4)	207 (26.2)	248 (31.4)				
	Yelling	176 (22.3)	554 (70.1)	60 (7.6)				
	Dog trainer, dog training school	173 (21.9)	244 (30.9)	373 (47.2)				
	Leash	52 (6.6)	263 (33.3)	475 (60.1)				
	Reward with play	31 (3.9)	151 (19.1)	608 (77)				
	Food	25 (3.2)	136 (17.2)	629 (79.6)				
	Petting	5 (0.6)	72 (9.1)	713 (90.3)				
	Praise	5 (0.6)	52 (6.6)	733 (92.8)				
**Problems in educating the dog**: what problem(s) have you had in raising your dog?		**Never (N**,** %)**	**Sometimes (N**,** %)**	**Often (N**,** %)**				
	Housing problems	735 (93)	42 (5.3)	13 (1.6)				
	Family problems	659 (83.4)	111 (14.1)	20 (2.5)				
	Financial problems	586 (74.2)	175 (22.2)	29 (3.7)				
	Problems to keep the dog while on vacations	580 (73.4)	167 (21.1)	43 (5.4)				
	Education started late	526 (66.6)	152 (19.2)	112 (14.2)				
	Socialisation problems (it was difficult to find company for the dog)	450 (57)	179 (22.7)	161 (20.4)				
	Lack of time	326 (41.3)	338 (42.8)	126 (15.9)				
	Inconsistency in rules/education	303 (38.4)	390 (49.4)	97 (12.3)				
	Impatience	220 (27.8)	476 (60.3)	94 (11.9)				
**Start of the dog’s education**: when did you start training your dog?					**N**			**%**
		Before 8 weeks old			61			7.7
		Between 8–12 weeks old			330			41.8
		Between 12–16 weeks old			101			12.8
		Between 4–6 months old			82			10.4
		Between 6–12 months old			68			8.6
		After 1 year old			148			18.7
**Housing conditions**: where do you keep the dog?		Indoors only			269			34.1
		Outdoor access			521			65.9
**Time spent in the presence of the dog**: how much time per day do you spend in the same airspace with your dog (so that your dog can come to you at any time)?		Less than 3 h			86			10.9
		3–9 h			222			28.1
		More than 9 h			482			61
**Active time spent with the dog**: exactly how much time a day do you spend actively with your dog? (walking, playing, etc.)		Less than 1 h			127			16.1
		1–3 h			543			68.7
		More than 3 h			120			15.2
*4) Dog-owner relationship*								
**Benefits derived from dog ownership**: how much do the following give you pleasure and a good feeling when living with your dog?		**None (N**,** %)**	**A little bit (N**,** %)**	**A lot (N**,** %)**				
	Safety, house guarding	296 (37.5)	278 (35.2)	216 (27.3)				
	Relationship with other people	192 (24.3)	311 (39.4)	287 (36.3)				
	Caring for someone, sense of responsibility	22 (2.8)	157 (19.9)	611 (77.3)				
	Teaching, training	13 (1.6)	162 (20.5)	615 (77.8)				
	The sight and beauty of the dog	13 (1.6)	79 (10)	698 (88.4)				
	Walking	1 (0.1)	106 (13.4)	683 (86.5)				
	Unconditional love	1 (0.1)	49 (6.2)	740 (93.7)				
	Petting, physical contact	1 (0.1)	18 (2.3)	771 (97.6)				
**Role of the dog**: what role does your dog play in your life?		**Not true at all (N**,** %)**	**Rather not true (N**,** %)**	**Neutral (N**,** %)**		**Rather true (N**,** %)**		**Totally true (N**,** %)**
	Assistance dog, guard-protection dog	450 (57)	87 (11)	107 (13.5)		94 (11.9)		52 (6.6)
	Colleague	443 (56.1)	91 (11.5)	126 (15.9)		79 (10)		51 (6.5)
	Child	250 (32)	96 (12)	153 (19)		151 (19)		140 (18)
	More important than any human	115 (15)	88 (11)	209 (26)		262 (33)		116 (15)
	Domesticated animal	96 (12.2)	78 (9.9)	168 (21.3)		148 (18.7)		300 (38)
	Friend	8 (1)	13 (1.6)	59 (7.5)		170 (21.5)		540 (68.4)
	Family member	1 (0.1)	1 (0.1)	24 (3)		94 (11.9)		670 (84.8)

More than half of the dogs in our sample were purebred dogs (64.4%). Ninety breeds were represented in total. Among purebred dogs, the most popular dog breed was the Border Collie (8.88%), followed by the Vizsla (5.92%), the Boxer (5.33%), the German Shepherd Dog (5.13%), the Labrador Retriever (5.13%), the Dachshund (4.14%) and the Belgian Sheperd Dog (4.14%). The other breeds were represented by 17 individuals or fewer (61.33% of other breeds). The mean age (± S.D.) of the dog in our sample was 5 years (± 3.6). Most dogs in our sample came from a registered breeder (42.7%) or from a shelter (32.9%). Four hundred twenty-four female dogs (98 intact) and 366 male dogs (120 intact) were counted.

### Questionnaire

The questionnaire consisted of four parts:

1. Characteristics of the owner.
2. Characteristics of the dog.
3. Management practices of the owner.
4. Dog-owner relationship.

Our questionnaire was originally developed in Hungarian, and it contained a total of 20 items. Only the name of the dog question and the last question (‘If there are any other behaviour problems your dog has or any positive things you would like to add to your dog’s life that we haven’t listed, or any comments you would like to make, please write them here’) were open-ended. However, open-text answers were not analysed in the present study. The English translation of the questionnaire can be found in the Supplementary materials (Supplementary Table S1).

1. *Characteristics of the owner*. We asked the owners about their (A) gender, (B) age (18–30/30–40/40–50/50–60/60–70/more than 70), and (C) whether they had children. Because participants over 60 years were rare in our sample, the three last age groups (50–60, 60–70 and more than 70) were merged together in a ‘more than 50’ group.
2. *Characteristics of the dog*. Owners were asked to indicate (A) the dog’s name, (B) the sex and neutering status of the dog, (C) if the dog was purebred, and if so, its breed. Because some owners did not specify the exact breed of the dog, some breeds/types were merged into broader categories, e.g., Dachshund (Miniature Dachshund, Wire-haired Dachshund, Long-haired Dachshund), Belgian Shepherd Dog (Malinois, Groenendael, Tervueren), Vizsla (Wire-haired Vizsla, Smooth-Haired Vizsla). We also asked about (D) the dog’s age, and (E) the source of the acquisition of the dog. The latest was recoded into a 3-level variable: (i) Dogs coming from a breeder: registered and non-registered breeder, puppy mill, (ii) Rescued dogs: shelter dogs, dogs found on the streets, dogs from dog breed rescuers, (iii) Other: dogs obtained through family/acquaintance, own breeding, other. Data about the behaviour of the dog were collected as well. Participants were asked to indicate E) how typical it was for their dog to display certain problematic behaviours (on a Likert scale from 1-not typical at all to 5-totally typical), F) how obedient they thought their dog was (on a Likert scale from 1-not at all to 5-totally), and G) how safe they felt with their dog off-leash (1-not at all to 5-totally). Because only a few participants rated their dog’s obedience a 1 or a 2, this variable was recoded into a 3-level variable by merging ratings 1, 2 and 3 together. The *off-leash safety* was recoded into a similar 3-level variable (see Table 1).
3. *Management practices of the owner*. Management practices comprise the training of the dog and keeping practices. We asked the owners about (A) the frequency of use of certain tools and methods to educate the dog, (B) the frequency of occurrence of certain problems they encountered (or not) during the dog’s education, (C) the age of the dog when they started its education, (D) the housing conditions of the dog, (E) the time spent in the presence of the dog per day, and (F) the active time spent with the dog per day (e.g., walking, playing). The original *housing conditions* variable included five categories: (1) only inside the house, (2) inside the house, but he/she can also go in the garden, (3) in the garden, but he/she can also go inside the house, (4) in the garden, (5) inside a kennel only/other. This variable was dichotomized: (1) only inside the house, (2) the dog has outdoor access. To simplify the interpretation of the results, the two *time spent with the dog* variables were recoded into 3-level variables. For the *active time spent with the dog per day*, the new categories were the following: (i) Less than an hour: owners who declared spending a few minutes or between 30 min and 1 h of activity with their dog per day, (ii) Between 1 and 3 h: owners who declared spending between 1 and 2 h or between 2 and 3 h of activity with their dog per day, (iii) More than 3 h: similar to the original category. For the t*ime spent in the presence of the dog per day*, the new categories were the following: (i) Less than 3 h: owners who declared spending less than 1 h or between 1 and 3 h in the presence of the dog per day, (ii) Between 3 and 9 h: owners who declared spending between 3 and 6 h or between 6 and 9 h in the presence of the dog per day, (iii) More than 9 h: owners who declared spending between 9 and 12 h or more than 12 h in the presence of the dog per day.
4. *Dog - owner relationship*. We asked about (A) how much pleasure owners have had with their dogs in relation to various typical benefits of dog ownership (on a 3-point Likert scale: None, A little bit, A lot), and (B) what role their dog was playing in their lives (on a 5-point Likert scale from 1-Not true at all to 5-Totally true). The roles we selected in the present study can be divided into two main categories:
  Animal-like (non-social) roles: assistance/guard-protection dog; domesticated animal.
  Human-like (social) roles: colleague, family member, friend, child.

As opposed to human-like roles, animal-like roles encompass those that refer specifically to the dog’s species and are not used to describe human relationships. The notion that the dog is a domesticated animal is a concept taught to all primary school students in Hungary. Therefore, when a dog owner deliberately refrains from referring to their dog with this term, it may indicate a profound psychological or emotional resistance to categorising the dog as an “animal”, reflecting the owner’s perception of their dog as a more humanised or unique entity. Finally, because intimacy and closeness also play important roles in defining a pet’s status, we included an eighth item to capture the centrality of the dog in the life of its owner, relative to other members of the owner’s social network: *more important than any human*.

### Statistical analysis

Statistical analysis was conducted using R.4.3.3 software.

Only one missing value was found for an item belonging to the *Problems in educating the dog* scale (‘Problems to keep the dog while on vacation’). It was replaced by the item’s median with the ‘replace’ function of ‘base’ package^42^.

Several items showed a highly skewed distribution (i.e., with an agreement rate > 80%), indicating low variability in the participants’ responses. This high skewness complicated the interpretation of the Multinomial Log-linear Model results, as certain response categories were either sparsely represented or completely absent across the three clusters. Insufficient data can lead to difficulties in parameter estimation and model interpretation. Therefore, the following items were kept in the descriptive analysis but not in further analyses. *Behaviour problems of the dog* scale: compulsive behaviour, escaping, house training problems. *Problems in educating the dog* scale: family problems, housing problems. *Tools and methods used to educate the dog* scale: praise, petting, muzzle, spiked collar, electric collar. *Benefits of having a dog* scale: walking, petting/physical contact, sight and beauty of the dog, unconditional love. *Other*: gender of the owner.

In order to determine different profiles of dog owners in our sample, we utilized the k-means clustering method on the *Role of the dog* scale. Data were first standardised with the ‘scale’ function of ‘base’ package^42^, and then we used the ‘NbClust’ package^43^ to determine the optimal number of cluster (Euclidean distance, kmeans method). After running the ‘kmeans’ function (‘stats’ package^44^) for the first time, a graphical representation of the clusters (using the ‘fviz_cluster’ function of ‘factoextra’ package^45^) highlighted three outliers presenting unusual patterns of response (i.e., rating most roles 1 and one or two roles 3 or a 5), who therefore were excluded from the final cluster analysis. A cluster was then assigned to each observation, and median ratings of the eight items were calculated for each cluster using the ‘aggregate’ function (‘stats’ package^44^). Median ratings of each role were compared between the three clusters using Kruskal-Wallis tests (‘kruskal.test’ function from ‘stats’ package^44^). Multiple pairwise comparisons between the three clusters were conducted using the ‘pairwise.wilcox.test’ function (‘stats’ package^44^).

A Principal Component Analysis (PCA) was conducted on each of the three following scales: *Tools and methods used to educate the dog*, *Behaviour problems of the dog*, *Problems in educating the dog*. These scales were ordinal, so first, a polychoric correlation matrix was created with the ‘polycor’ function of ‘mvord’ package^46^. A parallel analysis, using the ‘fa.parallel’ function of ‘psych’ package^47^, helped to determine the number of components to be extracted. PCA with an oblimin rotation was run with the ‘principal’ function of ‘psych’ package^47^. A cut-off value of 0.40 was used, and items that were not loading on any extracted component were removed from the final solution. Once all items were loaded on at least one component, factor scores were calculated with the ‘factor.scores’ function (tenBerge method) of ‘psych’ package. A Cronbach’s alpha was calculated for each extracted component. Only components with a Cronbach’s alpha > 0.60 were used in further analyses. As all the components obtained on the *Problems in educating the dog* scale had a low Cronbach’s alpha coefficient, we decided to include each item separately in the model building. PCA results for this specific scale are visible in Supplementary Table S2.

To analyse the effect of owner variables, dog variables, and owner management-related variables on the identified dog owner profiles, a Multinomial Log-Linear Model was used (‘multinom’ function from ‘nnet’ package^48^).

To find the most parsimonious model, we used a bottom-up, AIC-based model selection technique with two inclusion criteria: a likelihood ratio test with a p-value < 0.05, and at least two value differences between the models tested.

The most parsimonious model of Multinomial Log-linear Model for our outcome of interest *dog owner profile* contained the *use of professional training tools* as a covariate, and the *safety*,* house guarding benefit of having a dog*, the *age of the owner*, the *time spent in the presence of the dog*, the *dog’s housing conditions*, the *inconsistency in the dog’s education* problem, the *off-leash safety* and the perceived *obedience of the dog* as factors. However, it has to be noted that a model with a lower AIC value has been found, adding the *parental status of the owner* and the *problems to keep the dog while on vacation* to the other predictors. Because these additional variables did not meet our two inclusion criteria, they were not included in our final model.

In order to estimate the potential multicollinearity among the independent variables included in our final model, we measured the variance inflation factor (VIF) on three Binomial Generalized Linear Models (‘vif’ function from ‘car’ package^49^) for each cluster category, as VIF analysis cannot be applied to Multinomial Log-linear Models.

## Results

### Descriptives

#### Dog-owner relationship

The majority of our sample regarded their dogs as *family member*s (84.8% of the owners described this role as totally true) and as *friends* (68.4% of the owners described this role as totally true). *Domesticated animal* was the third most popular item to describe the dog’s role, rated as ‘totally true’ by 38% of the respondents. The *child* role, as well as the item *more important than any human*, divided the owners the most, with 15–32% of the owners declaring that these were ‘not true at all’ for their dogs and 15–18% declaring the opposite. The roles least used to describe the dog were *assistance/guard-protection dog* (57% declared that it was not true at all) and *colleague* (56.1% declared that it was not true at all).

What owners liked the most about living with their dog was, first, *petting and physical contact* (97.6%), followed by *unconditional love* (93.7%), the *sight and beauty of the dog* (88.4%), and *walking* (86.5%). Perceived benefits for which owners had more varied preferences were *safety and house guarding* (37.5% reported no benefit from it vs. 27.3% liked it ‘a lot’), and *relationship with other people* (24.3% reported no benefit from it vs. 36.3% liked it ‘a lot’).

#### Dog behaviour

The most prevalent behaviour problem in our sample was *jumping up* (33.2% of owners described it as typical or totally typical of their dog), *chasing wild/domesticated animals* (28.5% typical-totally typical of the dog), *territorial behaviour* (26.7% typical-totally typical of the dog) and *overexcitement* (20.9% typical-totally typical of the dog). Additionally, 24% of the owners rated their dog as *totally obedient*, and 30.6% as *totally safe off-leash*.

#### Dog management practices

As for training tools and methods, owners declared using *praising* (92.8%), *petting* (90.3%), and *food* (79.6%) the most. *Dog trainer and dog training school* were also popular solutions to train the dog, as 47.2% of the owners said that they often resort to them. On the other hand, owners stated that they have never used an *electric collar* (93.5%), a *spiked collar* (90.8%), and a *muzzle* (81.1%). Lastly, 62.5–66.6% of owners declared that they never used *physical discipline* nor *isolation* to educate their dogs, while *yelling* was indicated to be often used by 7.6% of owners.

Problems often encountered by owners to rear and educate their dog were *socialisation problems* (20.4%), *lack of time* (15.9%) and *late education* (14.2%). Conversely, most owners declared that they never encountered *housing problems* (93%), *family problems* (83.4%), or *financial problems* (74.2%) while raising their dogs. All descriptives results are reported in Table 1.

### Cluster analysis

#### Dog owner profiles

The k-means cluster analysis divided our sample of dog owners into three groups (Table 2), in regard to the roles they attributed to their dogs. Each dog owner was assigned to one of these three groups in the original dataset. Median ratings of each role significantly differed between groups (*p* < 0.001) except for the domesticated animal role (*p* = 0.395).

- Cluster 1 (N = 391, 49.5% of the sample), *dog parents*: dog owners who rated their dogs higher on the following roles: domesticated animal, friend, family member, child, and more important than any human, compared to the roles of colleague and assistance/guard dog. The label name emphasizes the close nature of the dog-owner relationship and the family-centered role of the dog, although not all of these roles referred to a ‘parent-child’ type relationship.
- Cluster 2 (*N* = 153, 19.4% of the sample), *companion dog* owners: they rated their dogs higher on the domesticated animal, friend, and family member roles in comparison to the four other roles. Additionally, median ratings for the friend and family member roles were significantly lower than those of the two other clusters (*p* < 0.001). While these owners seem to share the common Western view of dogs as companions who are part of the family, they appear to maintain a greater emotional distance from their dogs compared to the two other groups.
- Cluster 3 (*N* = 246, 31.1% of the sample), *dual status* owners: they agreed that their dog served all the presented roles, including the child role, the colleague role, and the assistance/guard dog role; therefore, they assigned a dual status (both practical and companionship functions) to their dogs.

**Table 2:** Median rating of each role of the dog within the three identified clusters of dog owners.

		Role of the dog (median rating)						
		Colleague	Assistance dog, guard dog	Domesticated animal	Friend	Family member	Child	More important than any human
**Dog owner clusters**	Cluster 1 (*Dog parents*), *N* = 391	1	1	4	5	5	3	4
	Cluster 2 (Regard dogs as *companion animals*), *N* = 153	1	1	4	4	4	1	2
	Cluster 3 (Assign dogs a *dual status*), *N* = 246	3	4	4	5	5	3	4

### Principal component analysis

#### Tools and methods used to educate the dog

Hygienic pads item was not kept in the PCA because they are mostly used to educate puppies and not adult dogs which comprised the majority of our sample. Parallel analysis suggested two principal components. The *leash* item did not reach the 0.40 cut-off on any component, so it was removed from the final model (Table 3).

**Table 3:** Results of the principal component analysis conducted on the *Tools and methods used to educate the dog* scale.

	Component loading	
Tool or method used to educate the dog	Positive reinforcement & professional training	Punitive methods
Eigenvalue	2.540	1.834
% of variance	31.8%	22.9%
Cronbach α	0.614	0.509
Clicker	**0.808**	− 0.098
Food	**0.796**	0.068
Dog trainer, dog training school	**0.753**	0.019
Reward with play	**0.579**	− 0.103
Cage/Room kennel	**0.564**	0.172
Physical discipline	− 0.045	**0.879**
Yelling	0.018	**0.871**
Isolation	0.173	**0.498**

The first component (α = 0.614) includes rewards (food, play) and tools (crate, clicker) usually used in conditioned positive reinforcement training approaches (association of a neutral stimulus with a reward), as well as training provided by professionals (i.e., dog trainers, dog school). The second component (α = 0.509) includes positive punishment methods (i.e., yelling, physical discipline) and negative punishment methods (i.e., isolation). Because of its low Cronbach’s α (< 0.60), this second component was not included in the model building. Only the component scores for the *Positive reinforcement & professional training* component were calculated and included in the model building.

#### Behaviour problems of the dog

Parallel analysis suggested three principal components for our model. PCA yielded into three components (Table 4), accounting for 54.7% of the total variance. The items *faeces eating/rolling in faeces* and *hard (or impossible) to call back during walks* did not reach the 0.40 cut-off on any component, so they were not included in the final model. Each of the three components includes behaviours frequently grouped together into bigger behaviour problem categories^29,50^, i.e., *fear* (α = 0.735), *excitability* (α = 0.662), and *aggressivity* (α = 0.617). Component scores were calculated for all components and included in the model building.

**Table 4:** Results of the principal component analysis conducted on the *Behaviour problems of the dog* scale.

	Factor loading		
Type of behaviour problem	Fear	Excitability	Aggressivity
Eigenvalue	2.816	2.678	2.392
% of variance	17.6%	16.8%	14.9%
Cronbach α	0.735	0.662	0.617
Fear of other dogs	**0.714**	0.007	0.115
Fear of other people	**0.769**	− 0.058	0.205
Fear of new things/situations	**0.828**	0.059	0.006
Noise phobia	**0.742**	0.013	− 0.136
Too much whining	0.324	**0.593**	− 0.117
Chewing/Destruction	− 0.063	**0.727**	− 0.058
Jumping up	0.049	**0.650**	− 0.051
Overexcitement	− 0.090	**0.773**	0.104
Too much barking	0.349	**0.446**	0.051
Separation anxiety	0.333	.**491**	− 0.049
Rough play/Pinching	− 0.187	**0.478**	**0.401**
Aggression to people	0.254	− 0.098	**0.713**
Aggression to other dogs	0.108	− 0.028	**0.698**
Territorial behaviour	− 0.068	0.003	**0.791**
Food/toy protection	0.033	0.222	**0.503**
Chasing wild/domesticated animals	− 0.096	0.203	**0.496**

### Determinants for the dog owner profile

VIF scores of the three Binomial Generalized Linear Models ranged from 1.08 to 1.93, indicating that no multicollinearity was found among our model’s independent variables.

The dog owner profile was found to be associated with the *age of the owner* (*p* = 0.001), the *off-leash safety* (*p* = 0.038), the perceived *obedience of the dog* (*p* < 0.001), the *dog’s housing conditions* (*p* < 0.001), the use of *positive reinforcement and professional training methods* (*p* = 0.006), the *inconsistency in the dog’s education* problem (*p* = 0.048), the *time spent in the presence of the dog* (*p* < 0.001) and the *safety benefit of having a dog* (*p* < 0.001). A summary of the main results obtained in the final model can be seen in Table 5. For a graphical summary of these results, please refer to Figure S1.

**Table 5:** Summary of the main results obtained in the final model (Multinomial Log-linear model). This table compares each cluster to the two other clusters.

		*Cluster 1* - Dog parents vs. other dog owners	*Cluster 2* - Regard dogs as companion animals vs. other dog owners	*Cluster 3* - Assign dogs a dual status vs. other dog owners
**1.** **Characteristics of the owner**	Age (*p* = 0.001)		Older (more likely to be 40 years old or above) than other owners	
	Owners did not significantly differ in the following variable: *parental status*			
**2.** **Characteristics of the dog**	Off-leash safety (*p* = 0.038)	Owners who regard dogs as companion animals were more likely to feel moderately safe with the dog off-leash compared to dog parents		
	Perceived obedience (*p* < 0.001)			More likely to have an obedient dog than other owners
	Owners did not significantly differ in the following variables: *sex of the dog; age of dog; source of acquisition of the dog; purebred status; age of the dog when training started; behaviour problems (fear*,* excitability*,* aggressivity)*			
**3.** **Management practices**	Housing of the dog (*p* < 0.001)	More likely to keep the dog indoors only than other owners		
	Use of positive reinforcement and professional training methods (*p* = 0.006)			More likely to use positive reinforcement and professional training than other owners
	Inconsistency in the dog’s education (*p* = 0.048)		Less likely to experience inconsistency in the dog’s education than other owners	
	Time spent in the presence of the dog (*p* < 0.001)		Less likely to spend at least 3 h/day in the presence of their dog than other owners	
	Owners did not significantly differ in the following variables: *active time spent with the dog; financial problems; problems to keep the dog while on vacation; education started late; socialisation problems; lack of time; impatience*			
**4.** **Dog-owner relationship**	Safety, house guarding benefit of having a dog (*p* < 0.001)			More likely to enjoy the safety/house guarding benefit of having a dog than other owners
	Owners did not significantly differ in the following variables: *relationship with other people; caring for someone*,* sense of responsibility; teaching*,* training*			

#### Characteristics of the owner

**Fig. 1:**
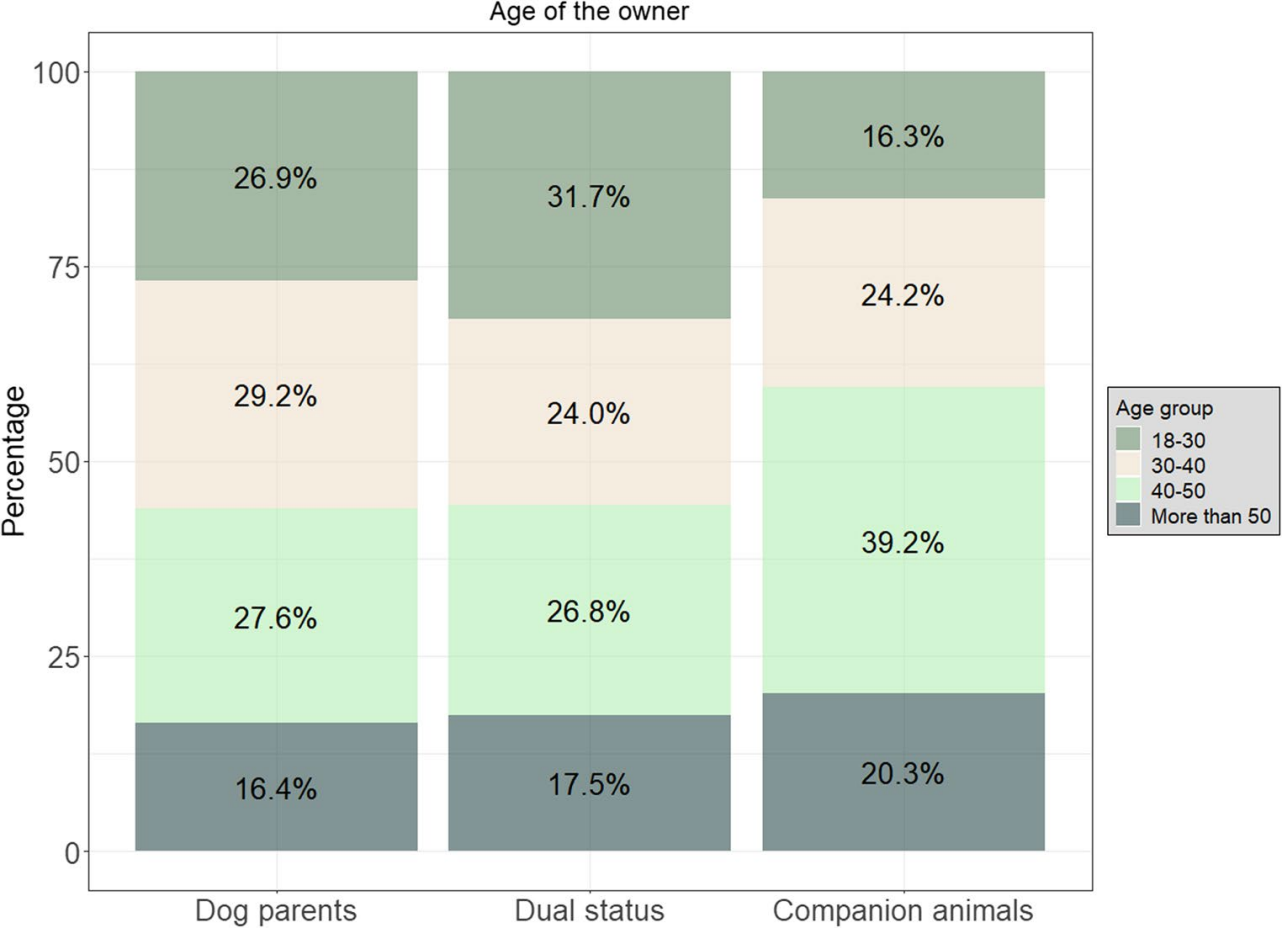
Differences between the dog owner profiles regarding the characteristics of the owner. In our model, age of the owner was the only characteristic significantly different between dog owner profiles.

The only demographic variable we found to be associated with the dog owner profile was the age of the owner. Owners of companion dogs were older than owners of dogs with a dual status (*30–40* vs. *18–30 (ref)*: ß ± SE: 0.86 ± 0.33; Z = 2.58; OR = 2.36 [1.23–4.55]; *p* = 0.01, *40–50* vs. *18–30 (ref)*: ß ± SE: 1.32 ± 0.32; Z = 2.96; OR = 3.75 [2.02–6.99]; *p* < 0.001, *more than 50* vs. *18–30 (ref)*: ß ± SE: 1.08 ± 0.36; Z = 2.96; OR = 2.95 [1.44–6.04]; *p* = 0.003) and than dog parents (*40–50* vs. *18–30 (ref)*: ß ± SE: 0.91 ± 0.29; Z = 3.12 ; OR = 2.5 [1.40–4.43]; *p* = 0.002, *more than 50* vs. *18–30 (ref)*: ß ± SE: 0.73 ± 0.34; Z = 2.16; OR = 2.08 [1.07–4.03]; *p* = 0.03). Compared to dog parents, owners of dogs with a dual status were more likely to be younger than 30 years old than to be aged between 30 and 40 years old (*30–40* vs. *18–30 (ref)*: ß ± SE: -0.51 ± 0.24; Z =-2.15; OR = 0.60 [0.37–0.95]; *p* = 0.03). No significant differences were found between the other age groups between dog parents and owners of a dog with a dual status. (Fig. 1).

#### Characteristics of the dog

**Fig. 2:**
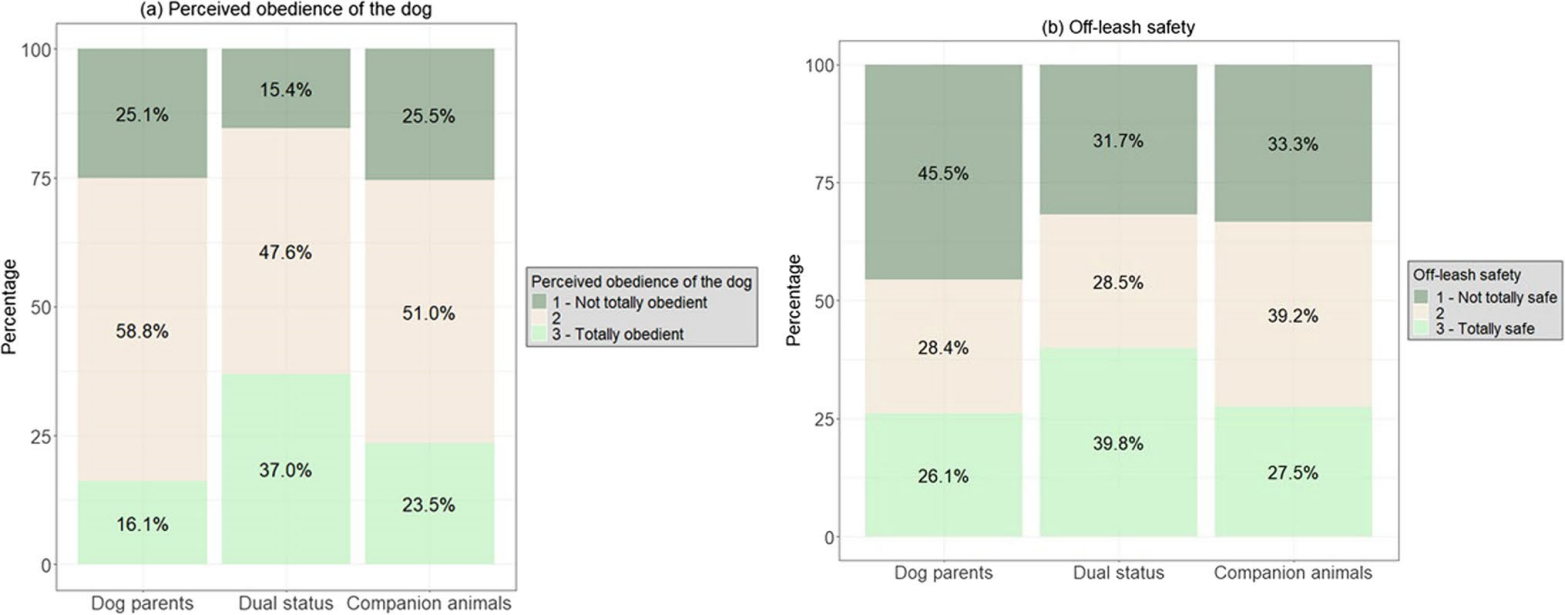
Differences between the dog owner profiles regarding the characteristics of the dog: (**a**) the perceived obedience of the dog and (**b**) the off-leash safety.

Two behavioural factors were found to be associated with the dog owner profiles. First, owners who assigned a dual status to their dog were more likely to report a more obedient dog in comparison to dog parents (*3* vs. *1 (ref)*: ß ± SE: 1.09 ± 0.33; Z = 3.31; OR = 3 [1.56–5.64]; *p* < 0.001, *3* vs. *2 (ref)*: ß ± SE: 0.94 ± 0.24; Z = 3.89; OR = 2.6 [1.60–4.14]; *p* < 0.001) and to owners keeping dogs as companion animals (*3* vs. *1 (ref)*: ß ± SE: 0.95 ± 0.41; Z = 2.32; OR = 2.58 [1.16–5.76]; *p* = 0.02) (Fig. 2a).

Regarding the off-leash safety, owners of a companion dog were more likely to feel moderately safe with their dog off-leash compared to dog parents (*2* vs. *1 (ref)*: ß ± SE: 0.73 ± 0.26; Z = 2.80; OR = 2.1 [1.25–3.47]; *p* = 0.005, *2* vs. *3 (ref)*: ß ± SE: 0.58 ± 0.29; Z = 1.99; OR = 1.78 [1.00-3.16]; *p* = 0.046) (Fig. 2b).

Although the dog’s specific breed was not included in our model (as we had many mixed-breed dogs in our sample), we also compared the five most frequently owned breeds in each cluster. The most popular breeds in the ‘dog parents’ cluster were the Border Collie (representing 8.9% of the purebred dogs from this cluster), the Vizsla (6.8%), the Boxer (6.4%), the Dachshund (6.4%) and the Pumi (4.3%). In the ‘companion animals’ cluster, the Mudi (7.1%), the English Cocker Spaniel (6.1%), the Labrador Retriever (6.1%), the Boxer (5.1%) and the German Sheperd Dog (5.1%) were the most represented. Finally, in the last cluster (‘dual status’), popular breeds were the Border Collie (11.5%), the Belgian Sheperd Dog (8%), the German Sheperd Dog (7.5%), the Labrador Retriever (6.3%) and the Vizsla (6.3%).

#### Management practices of the owner

**Fig. 3:**
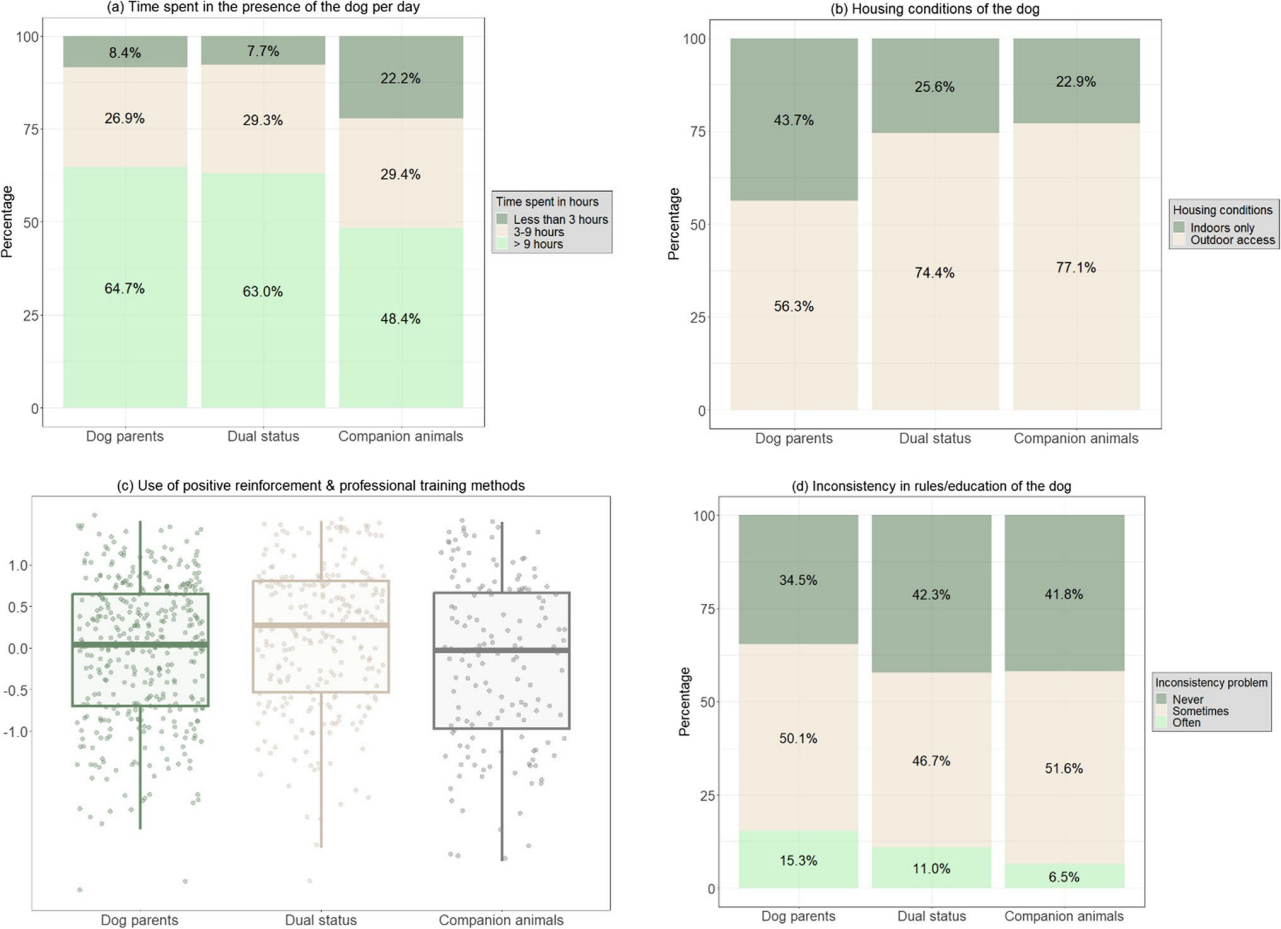
Differences between the dog owner profiles regarding the management practices of the owner: (**a**) the time spent in the presence of the dog per day, (**b**) the housing conditions of the dog, (c) the use of positive reinforcement and professional training methods, and (d) the inconsistency in rules/education of the dog.

Regarding the management practices, first, we found that dog parents were more likely to house their dog indoors only compared to owners whose dogs have a dual status (ß ± SE: 0.77 ± 0.20; Z = 3.87; OR = 2.2 [1.46–3.19]; *p* < 0.001) and to owners of a companion dog (ß ± SE: 0.73 ± 0.24; Z = 3.06; OR = 2.07 [1.30–3.31]; *p* = 0.002) (Fig. 3a).

Moreover, dog parents spent more time in the presence of their dog per day than owners of companion dogs (*3–9 h* vs. *< 3 h (ref)*: ß ± SE: 0.73 ± 0.33; Z = 2.22; OR = 2.1 [1.09–3.98]; *p* = 0.03, *> 9 h* vs. *< 3 h (ref)*: ß ± SE: 1.24 ± 0.32; Z = 3.92; OR = 3.5 [1.86–6.42]; *p* < 0.001, *> 9 h* vs. *3–9 h* (ref): ß ± SE: 0.50 ± 0.24; Z = 2.14; OR = 1.66 [1.04–2.63]; *p* = 0.03). Similarly, owners of a dog with a dual status spent more time in the presence of their dog per day than owners of companion dogs (*3–9 h* vs. *< 3 h (ref)*: ß ± SE: 1.24 ± 0.38; Z = 3.27; OR = 3.4 [1.64–7.23]; *p* = 0.001, *> 9 h* vs. *< 3 h (ref)*: ß ± SE: 1.77 ± 0.36; Z = 4.88; OR = 5.9 [2.89–12.01]; *p* < 0.001, *> 9 h* vs. *3–9 h* (ref): ß ± SE: 0.54 ± 0.26; Z = 2.08; OR = 1.71 [1.03–2.83]; *p* = 0.04) (Fig. 3b).

Focusing on training practices, according to our model, owners of dogs with a dual status were more likely to declare using positive reinforcement and professional training methods than dog parents (ß ± SE: 0.28 ± 0.11; Z = 2.60; OR = 1.32 [1.07–1.63]; *p* = 0.009) and than owners of companion dogs (ß ± SE: 0.37 ± 0.13; Z = 2.89; OR = 1.5 [1.13–1.88]; *p* = 0.004) (Fig. 3c). Secondly, regarding the problems encountered during the dog’s education, owners of companion dogs were less likely to experience inconsistency in rules/education compared to dog parents (*never* vs. *often (ref)*: ß ± SE: 1.10 ± 0.40; Z = 2.76; OR = 3 [1.38–6.57]; *p* = 0.006, *sometimes* vs. *often (ref)*: ß ± SE: 0.97 ± 0.38; Z = 2.54; OR = 2.6 [1.25–5.61]; *p* = 0.01) and to owners of a dog with a dual status (*never* vs. *often (ref)*: ß ± SE: 1.04 ± 0.44; Z = 2.38; OR = 2.83 [1.20–6.67]; *p* = 0.02, *sometimes* vs. *often (ref)*: ß ± SE: 0.97 ± 0.42; Z = 2.29; OR = 2.6 [1.15–6.04]; *p* = 0.02) (Fig. 3d).

#### Dog-owner relationship

**Fig. 4:**
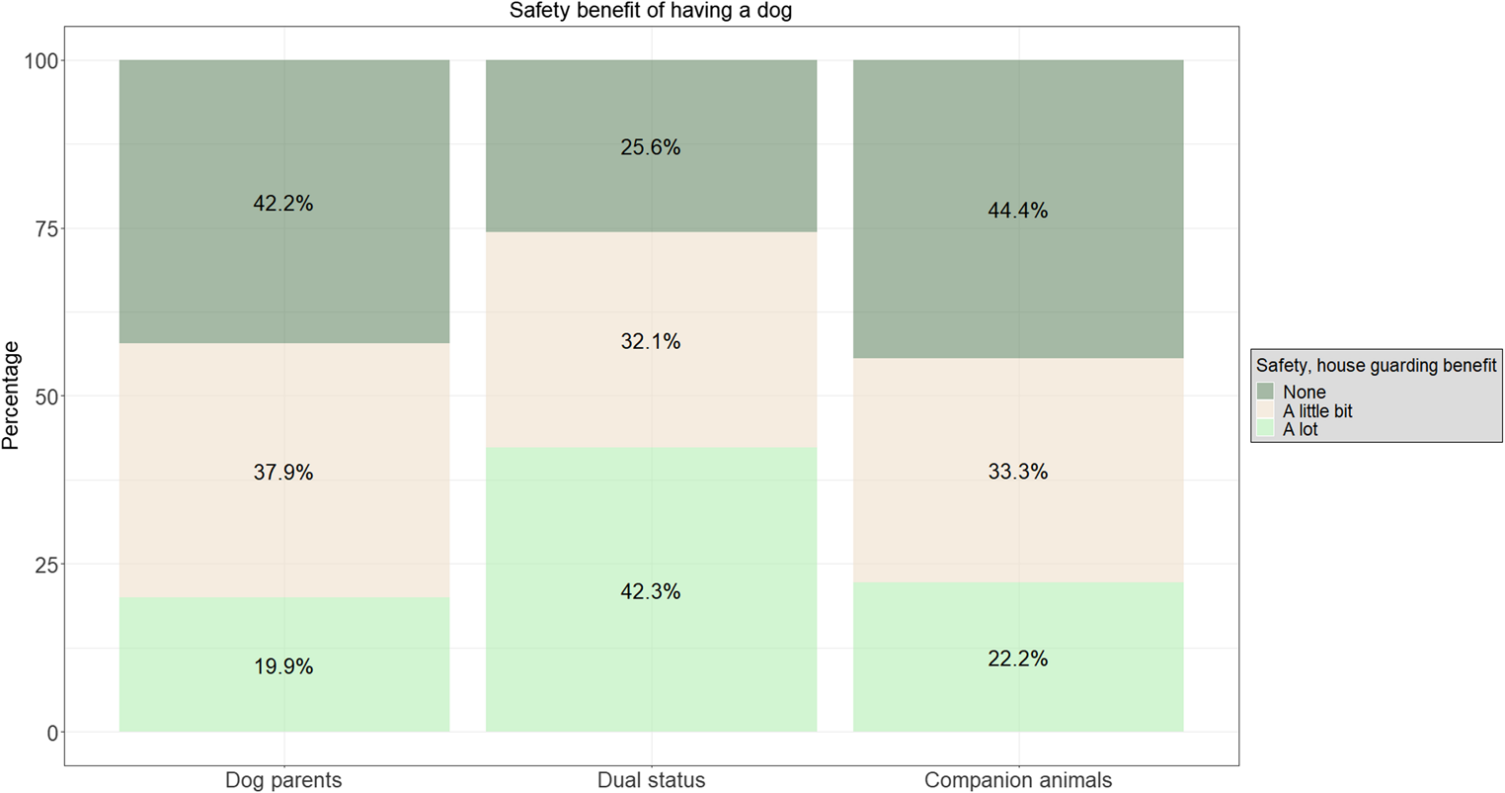
Differences between the dog owner profiles regarding the dog-owner relationship. In our model, the safety/house guarding benefit of having a dog was the only factor significantly different between dog owner profiles.

Owners assigning a dual status to their dog were more likely to find a safety benefit in having a dog compared to dog parents (*much* vs. *not at all (ref)*: ß ± SE: 1.37 ± 0.23; Z = 5.88; OR = 3.92 [2.49–6.19]; *p* < 0.001, *much* vs. *a little (ref)*: ß ± SE: 1.04 ± 0.22; Z = 4.70; OR = 2.83 [1.83–4.36]; *p* < 0.001) and to owners of companion dogs (*much* vs. *not at all (ref)*: ß ± SE: 1.83 ± 0.30; Z = 6.08; OR = 6.2 [3.45–11.18]; *p* < 0.001, *a little* vs. *not at all (ref)*: ß ± SE: 0.69 ± 0.27; Z = 2.58; OR = 2 [1.18–3.37]; *p* = 0.01, *much* vs. *a little (ref)*: ß ± SE: 1.14 ± 0.29; Z = 3.86; OR = 3.1 [1.75–5.55]; *p* < 0.001) (Fig. 4).

## Discussion

Our aim was to investigate the relationship between dogs’ roles, owner and dog characteristics, including behaviour problems, management practices and benefits derived from dog ownership. As expected, our analysis divided the dog owners in our sample into three distinct profiles associated with specific owner variables, dog variables and management-related variables. Nevertheless, most dog owners in our study agreed upon several aspects of dog ownership. They declared benefiting greatly from the unconditional love and physical contact provided by their dogs, and they found the sight and beauty of their dogs very enjoyable. Moreover, similar to American dog owners surveyed in 2021^51^, they report a clear preference for certain training tools (e.g., food, leash), while they avoid electric collars and spiked collars.

In accordance with our first hypothesis and with previous studies investigating the roles of dogs, a large proportion of dog owners from our convenience sample perceived their dog as a *family member*. This was also found to be the case in a representative sample of Hungarian dog owners, in which 66% of them agreed (slightly or completely) that their dog was a family member^6^. Another social role was similarly predominant: the dog as a *friend*. Because they share their daily lives together, it is likely that the dog and its owner develop a close, enduring social bond involving cooperative interactions, or in other words, a relationship analogous to human friendship. Indeed, such bonds are not restricted to human interactions, as they have been commonly described in many animal species^52^. Lastly, and perhaps not so surprisingly, a non-social role was preponderant in our sample: the dog as a *domesticated animal*. Even if they attribute social roles to dogs, it is likely that many people are willing to acknowledge that dogs belong to a different species, as previously suggested by^37,53^. Alternatively, since the first fact children learn about dogs in primary school is that they are domesticated animals, they may simply have reiterated this knowledge.

Despite the general trend in the roles attributed to the dogs in our sample, three distinct profiles of dog owners were identified. Owners in one of them regarded dogs primarily as family members, friends and domesticated animals. We also identified two distinct profiles of owners who viewed their dogs as fulfilling ‘more’ than these three roles. In these two profiles, high median ratings on the *child* and *more important than any human* items suggested that the dog-owner bond might be perceived as a physically and emotionally close, intimate relationship, although potentially unequal^37^. Owners of one of these two profiles additionally perceived their dog as a *colleague* and *assistance/guard dog*, illustrating the different functions that dogs can perform simultaneously, whether social or practical, and similarly to the complex relationship existing between people and their assistance dogs^54,55^.

As hypothesised, different variables were associated with the perceived roles of the dog in our model. First, we found that older owners were more likely to see their dog as a companion animal. Owners belonging to this cluster rated all social roles of the dog lower than other owners and agreed less that their dog was more important than any human, suggesting less emotional closeness to the dog. This is in line with findings reported by Dotson and Hyatt^56^, which indicated that dog owners under the age of 35 had higher levels of symbiotic relationship (characterised by a high affective involvement with the dog and more effort put into the dog’s care), whereas dog owners over the age of 65 scored the lowest on the anthropomorphic dimension (understood here as perceiving the dog as more of a human child and family member, and less as an animal). Thus, our result might reflect a generational shift in the perception of the dog-owner relationship, as observed in other cultures, too^57,58^.

The finding that younger owners were less likely to fall in the ‘dogs as companion animal’ cluster might indicate that they are more likely to see their pets as children, consistent with the literature^53,59–63^. However, our results cannot conclude on a relationship between parental status and the role of the dog, as this variable did not meet our two model inclusion criteria. The link between age, parental status and role of the dog should be explored further in future studies.

Compared to owners of companion animals, the two profiles of owners for whom the dog was also a *child* and *more important than any human* experienced more inconsistency in the dog’s education, meaning that they don’t provide a consistent response (e.g., praising, behaviour correction) every time the dog displays a specific behaviour. As they were also younger, one hypothesis is that these owners were more likely to be first-time owners. Indeed, compared to experienced owners, first-time owners might be more uncertain and less confident in their abilities to raise and train a dog^64^. Another potential explanation would relate to the average size of dogs kept in each cluster, as owners of smaller dogs have been found to be more inconsistent than owners of larger dogs^65^. Since small dogs might be more prone to being regarded as children (Gillet and Kubinyi, in preparation), it would be interesting to take this variable into account in future work to better understand the relationship between the size of the dog, the role of the dog, and the owner’s caring behaviour. Likewise, compared to owners of companion dogs, dog parents were more likely to feel unsafe with their dogs off-leash. It is possible that regarding the dog as a child increases the occurrence of overprotective behaviour (Gillet and Kubinyi, in preparation), which in this case could be associated with a greater need to control the dog’s behaviour during walks.

Owners attributing a dual status to their dog showed an interesting profile. On one hand, they kept their dog for utilitarian purposes. They enjoyed the safety provided by their dog more than other owners, and their dogs were assigned an *assistant/guard dog* role, which was not present in the two other profiles. Additionally, the high median rating of the *colleague* role of the dog suggested that they shared some type of work relationship with their dog. Owners of these ‘dual status dogs’ were likely to perceive their dog as more obedient than other owners. This could be connected to keeping a dog for work purposes, for which a specific dog and dog breed might be required, although the purebred status was not significantly associated with a dog owner profile in our model. It should be noted, however, that the four most popular breeds in this group (i.e., Border Collie, Belgian Sheperd Dog, German Sheperd Dog, and Labrador Retriever) are known for their working and training abilities^66^, suggesting that these owners, at least those choosing to acquire a purebred dog, might indeed turn to specific breeds. Working dogs have been shown to display different behavioural traits than companion dogs, including higher trainability and lower fearfulness^67,68^. Our finding is also in line with the observation reported by Kubinyi et al.^33^, who found that dogs kept as family members were less trainable and less calm than family dogs kept with other specific functions, such as work or guarding.

The fact that these owners were also more likely to declare using positive reinforcement and professional training methods suggests that they might put more effort into training their dogs so that they are able to perform various work tasks. Reward-based training methods (e.g., using treats and praising) have been described as ‘dog-centric’, taking into account the needs of the dog as much as the needs of the human. Therefore, their use may have a positive impact on canine welfare, unlike training methods based on a dominance-submission approach^69,70^. The use of reward-based training methods was also found to be positively correlated to the perceived obedience of the dog^70^. All in all, this type of positive training might contribute to improving and reinforcing the dog-owner relationship^69,71^, which in this group of owners may be reflected by the dual status of the dog.

Indeed, in addition to the previously described practical functions of the dog, owners of ‘dual status dogs’ were also characterised by a high median rating on the *child* and *more important than any human* items. Similarly to dog parents, they spent more time in the presence of their dog per day in comparison to owners of companion dogs. This could contribute to the development of a more intimate bond, as previously described in the literature^56^. One hypothesis is that this increased time spent with the dog is due to the multiple functions of these ‘dual status dogs’, who might accompany their owner during work time but also for off-time activities. That being said, it should be noted that we did not find any relationship between the active time spent with the dog and the dog owner profiles. This suggests that, despite spending less time in the presence of their dogs compared to other owners, owners of companion dogs appeared to invest just as much time actively caring for their dogs.

As for dog parents, one reason why they spend more time in the presence of their dogs compared to owners of companion animals might be connected to the housing conditions of the dog. Indeed, we found that, compared to the other groups of owners, dog parents were more likely to keep the dog indoors only. Although we did not collect data on the owners’ place of living in our study, this might mean that they were also more likely to live in urban environments. Baranyiová et al.^72^ argue that urban dogs are mostly kept for companionship and that by living in a smaller, more restrictive space (e.g., city flats), they are more dependent on humans and share more intimate bonds with them. Research has also shown that human contact is important for canine well-being^73,74^, and that social isolation can lead to separation anxiety^75^, a behaviour disorder with various (undesirable) manifestations that often lead to the dog’s relinquishment or euthanasia. Nonetheless, in our sample’s case, the perception of dog-owner relationship as a closer, more intimate bond was connected to greater time spent in the presence of the dog, but not with behaviour problems such as fear or separation anxiety, therefore suggesting a possible positive impact on canine welfare. This is also in line with the results reported by Kobelt et al.^76^, who have observed a negative correlation between the time spent with the dog and the dog’s excitability.

Altogether, our results did not reveal any problematic behaviour associated with the roles attributed to dogs. One possible explanation is that owners from our convenience sample could have been more prone to perceive their relationship with their dog in terms of close, lasting social bonds because their dogs did not display any problematic behaviour to start with^34,50^.

Another hypothesis could be that attributing human-like roles to dogs is different from having anthropomorphic attitudes towards them. By assuming that dogs possess human-like emotions and cognitive abilities (i.e., anthropomorphism), owners may potentially misinterpret their dog’s behaviour^25,28,58,77^, which could have negative consequences on dogs’ emotional states and behaviours. Yet, describing the dog-owner relationship in human terms (e.g., child, friend, colleague) may not always be associated with anthropomorphic attitudes towards dogs, as these terms can be used as analogies to convey the strong emotional bond between the two parties^37,78^. The fact that all owner groups in our sample perceived their dog as a domesticated animal supports this hypothesis: while owners all share a (more or less) close bond with their dogs, they still recognise and treat their dogs as a different species with distinct needs.

It should be noted, however, that as owners choose certain words to describe their relationship with their dog based on what they know from their human relationships, not all owners necessarily put the same meaning behind these terms. For instance, not everyone has internalized the same working models of friendships^79^, and therefore does not expect or appreciate the same things in a friend. Moreover, it can be argued that ‘family member’ can designate both a member of the nuclear family as well as a distant relative.

### Limitations

Since our participants were not representative of the general dog owning population (as they were mostly young, childless women), the generalisation of the above-mentioned results is limited. For instance, we did not find a group of owners for whom dogs fulfilled non-social roles and/or practical functions only. This could be because attitudes towards dogs and the perception of the dog-owner relationship can be influenced by demographic variables, such as gender. Specifically, several studies reported that women are more likely than men to be emotionally invested in their relationship with their dogs and to display nurturing behaviours towards them^56,80,81^. Gender can influence dog management practices as well. For example, female dog trainers have been found to use positive reinforcement more often than their male counterparts^82^.

Additionally, while disseminating our questionnaire in our social media groups enables us to reach thousands of dog owners, the drawback of this data collection method is that these respondents are also likely to be interested in dogs and canine ethology. Therefore, they might be more knowledgeable about dog management and more inclined to use certain training methods compared to the general population of dog owners^83^. Conversely, dog owners rarely engaging in activities with their dogs and using aversive training methods are usually difficult to access in questionnaire studies. All of these reasons might explain, at least partly, the high agreement rates obtained on certain items.

High agreement rates could also be due to the use of self-reported questions. Indeed, some respondents might have been reluctant to admit using certain training methods or having problems with their dogs, to conform with perceived social norms and present themselves as ‘responsible’, ‘good’ owners^83,84^. Research also found that people tend to overestimate their pets’ qualities^85^. This pet-enhancement bias could have affected the owners’ responses regarding their dogs. For instance, they may have underestimated their dogs’ behavioural problems or perceived their dogs as more obedient than they actually were.

Other limitations due to construct measurements, are, first, our use of several single-item measures to assess the dog’s behaviour. They were preferred in order to keep our questionnaire short and increase the participation rate, despite a risk for decreased predictive validity compared to multi-item measures. For the same reason, we asked the owners a limited number of demographic questions, which may limit the interpretation of some of our results. Additionally, it can be argued that the use of a 3-point scale over a 5-point scale in several questions may have contributed to the low variability observed in our sample.

## Conclusion

Despite the general trend observable in Western countries, in which dogs are more and more perceived as family members providing unconditional love and support, this study highlights that not all dog owners are the same, even in a convenience sample interested in dog behavioural studies. Our results show that dogs can fill multiple (social and non-social) roles simultaneously in their owners’ lives, highlighting the complexity of the dog-human relationship. Our findings also suggest that these roles are associated with different dog and owner characteristics. Importantly, attributing utilitarian, non-social roles to dogs, together with human-like roles, does not mean that dogs occupy a peripheral place in humans’ social circles. The dog can be perceived as a colleague, a friend, and the most important being in its owner’s life. Moreover, keeping a dog for useful purposes (e.g., assistance, safety) and not for company only is not a synonym for lowered care. Our study did not identify any major welfare concerns in relation to the roles attributed to dogs, as variations in roles and in management practices were not connected to behaviour problems. An association with behaviour problems could have indicated that these management practices were inappropriate, thus compromising the dog’s welfare. On the contrary, and similarly to previous findings^35,63^, we found that attributing multiple functions to dogs may enhance the time and effort put into the dog’s care, which in turn could improve the dog’s perceived obedience.

Although, as mentioned earlier, our results may not be generalisable to the entire owned dog population, we believe that they contribute to a better understanding of the modern dog-human relationship. The perception of this dog-owner relationship, described in terms of multiple roles coexisting together, remains an understudied topic, especially when investigating how owners care for their dogs and its consequences in terms of canine welfare. Thus, our new methodological approach lays the groundwork for future work aiming to investigate the various roles occupied by domesticated animals in society. Further research should continue to investigate this topic and take an interest in the connections between dogs’ social roles, anthropomorphism, management practices and outcomes for the dogs.

## Supplementary Information

Below is the link to the electronic supplementary material.

Supplementary Material 1

## Data Availability

The datasets generated and analysed during the current study are available from the corresponding author upon reasonable request.
