# Supplementary material for: The role of dogs is associated with owner management practices and characteristics, but not with perceived canine behaviour problems
Source: Sci Rep. 2024 Nov 12;14:27548. doi: 10.1038/s41598-024-77400-y (PMC11557872; doi:10.1038/s41598-024-77400-y)
Supplement: Supplementary file 1 — Supplementary Material 1 [file 41598_2024_77400_MOESM1_ESM.docx]

**Supplementary Table S1**

Original questionnaire (English translation)

1. Name of the dog: ______________
2. Age of the dog (in years): _____________
3. Sex of the dog:

- Neutered female
- Neutered male
- Intact female
- Intact male

1. Breed of the dog

- Mix breed
- Purebred: _____________

1. Gender of the owner:
   - Woman
   - Man
2. Age of the owner:
   - 18-30
   - 30-40
   - 40-50
   - 50-60
   - 60-70
   - >70
3. Do you have children?
   - No
   - Yes
4. How obedient do you think your dog is?

1 = Not at all to 5 = Totally

1. What tools/methods do you use to train your dog?

|  | **Never** | **I have used it before** | **Often** |
| --- | --- | --- | --- |
| Cage/Room kennel |  |  |  |
| Clicker |  |  |  |
| Dog trainer, dog training school |  |  |  |
| Electric collar |  |  |  |
| Food |  |  |  |
| Isolation |  |  |  |
| Leash |  |  |  |
| Muzzle |  |  |  |
| Petting |  |  |  |
| Physical discipline |  |  |  |
| Praise |  |  |  |
| Reward with play |  |  |  |
| Spiked collar |  |  |  |
| Training hygienic pads |  |  |  |
| Yelling |  |  |  |

1. When did you start training your dog?
   - Before 8 weeks old
   - Between 8–12 weeks old
   - Between 12–16 weeks old
   - Between 4–6 months old
   - Between 6–12 months old
   - After 1 year old
2. Where did you buy/adopt your dog?
   - From a breeder
   - From a shelter
   - From family/acquaintance
   - I found her/him on the streets
   - From a puppy mill
   - Other:
3. How safe do you feel taking the leash off your dog on the street/public space/forest?

1 = Not at all to 5 = Totally

1. How much time per day do you spend in the same airspace with your dog (so that your dog can come to you at any time)?
   - Less than 1 hour
   - 1–3 hours
   - 3–6 hours
   - 6–9 hours
   - 9–12 hours
   - More than 12 hours/all day
2. Where do you keep the dog?
   - Only inside the house
   - Inside the house, but he/she can also go in the garden
   - In the garden, but he/she can also go inside the house
   - In the garden/inside a kennel only
   - Other
3. Exactly how much time a day do you spend actively with your dog? (walking, playing, etc.)
   - A few minutes
   - Less than 30 minutes
   - 30 minutes–1 hour
   - 1–2 hours
   - 2–3 hours
   - More than 3 hours
4. What role does your dog play in your life?

|  | **Not true at all** | **Rather not true** | **Neutral** | **Rather true** | **Totally true** |
| --- | --- | --- | --- | --- | --- |
| Colleague |  |  |  |  |  |
| Assistance dog, guard-protection dog |  |  |  |  |  |
| Domestic animal |  |  |  |  |  |
| Friend |  |  |  |  |  |
| Family member |  |  |  |  |  |
| Child |  |  |  |  |  |
| More important than any human |  |  |  |  |  |

1. How typical are the following behaviour problems for your dog?

|  | **Not typical at all** | **Not typical** | **I don’t know/I can’t decide/Neutral** | **Typical** | **Totally typical** |
| --- | --- | --- | --- | --- | --- |
| Aggression to people |  |  |  |  |  |
| Aggression to other dogs |  |  |  |  |  |
| Fear of other dogs |  |  |  |  |  |
| Fear of other people |  |  |  |  |  |
| Fear of new things/situations |  |  |  |  |  |
| Noise phobia |  |  |  |  |  |
| Too much barking |  |  |  |  |  |
| Too much whining |  |  |  |  |  |
| Chewing/Destruction |  |  |  |  |  |
| Jumping up |  |  |  |  |  |
| House training problems |  |  |  |  |  |
| Escaping |  |  |  |  |  |
| Faeces eating/Rolling in faeces |  |  |  |  |  |
| Rough play/Pinching |  |  |  |  |  |
| Food/toy protection |  |  |  |  |  |
| Territorial behavior |  |  |  |  |  |
| Overexcitement |  |  |  |  |  |
| Separation anxiety |  |  |  |  |  |
| Hard (or impossible) to call back during walks |  |  |  |  |  |
| Compulsive behavior (e.g. circling) |  |  |  |  |  |
| Chasing wild/domesticated animals |  |  |  |  |  |

1. What problem(s) have you had in raising your dog?

|  | **Never** | **Sometimes** | **Often** |
| --- | --- | --- | --- |
| Impatience |  |  |  |
| Inconsistency in rules/education |  |  |  |
| Education started late |  |  |  |
| Socialisation problems (it was difficult to find company for the dog) |  |  |  |
| Lack of time |  |  |  |
| Financial problems |  |  |  |
| Housing problems |  |  |  |
| Family problems |  |  |  |
| Problems to keep the dog while on vacations |  |  |  |

1. How much do the following give you pleasure and a good feeling when living with your dog?

|  | **None** | **A little bit** | **A lot** |
| --- | --- | --- | --- |
| Walking |  |  |  |
| Petting, physical contact |  |  |  |
| Safety, house guarding |  |  |  |
| The sight and beauty of the dog |  |  |  |
| Teaching, training |  |  |  |
| Unconditional love |  |  |  |
| Caring for someone, sense of responsibility |  |  |  |
| Relationship with other people |  |  |  |

1. If there are any other behaviour problems your dog has or any positive things you would like to add to your dog's life that we haven't listed, or any comments you would like to make, please write them here: ___________________

| **Supplementary Table S2**  Results of the Principal Component Analysis conducted on the *Problems in raising the dog* scale | | | |
| --- | --- | --- | --- |
|  | **Factor loading** | | |
| **Type of problem encountered during the dog’s education** | **Practical problems** | **Dog-related problems** | **Owner-related problems** |
| Eigenvalue | 1.471 | 1.462 | 1.471 |
| % of variance | 21% | 20.9% | 20.6% |
| Cronbach α | .407 | .483 | .466 |
| Financial problems | **.846** | -.025 | -.095 |
| Lack of time | **.613** | .055 | .275 |
| Problems to keep the dog while on vacations | **.569** | .209 | .040 |
| Education started late | -.029 | **.832** | .061 |
| Socialisation problems (it was difficult to find company for the dog) | .068 | **.836** | -.061 |
| Inconsistency in rules/education | -.135 | .105 | **.850** |
| Impatience | .177 | -.114 | **.789** |

**
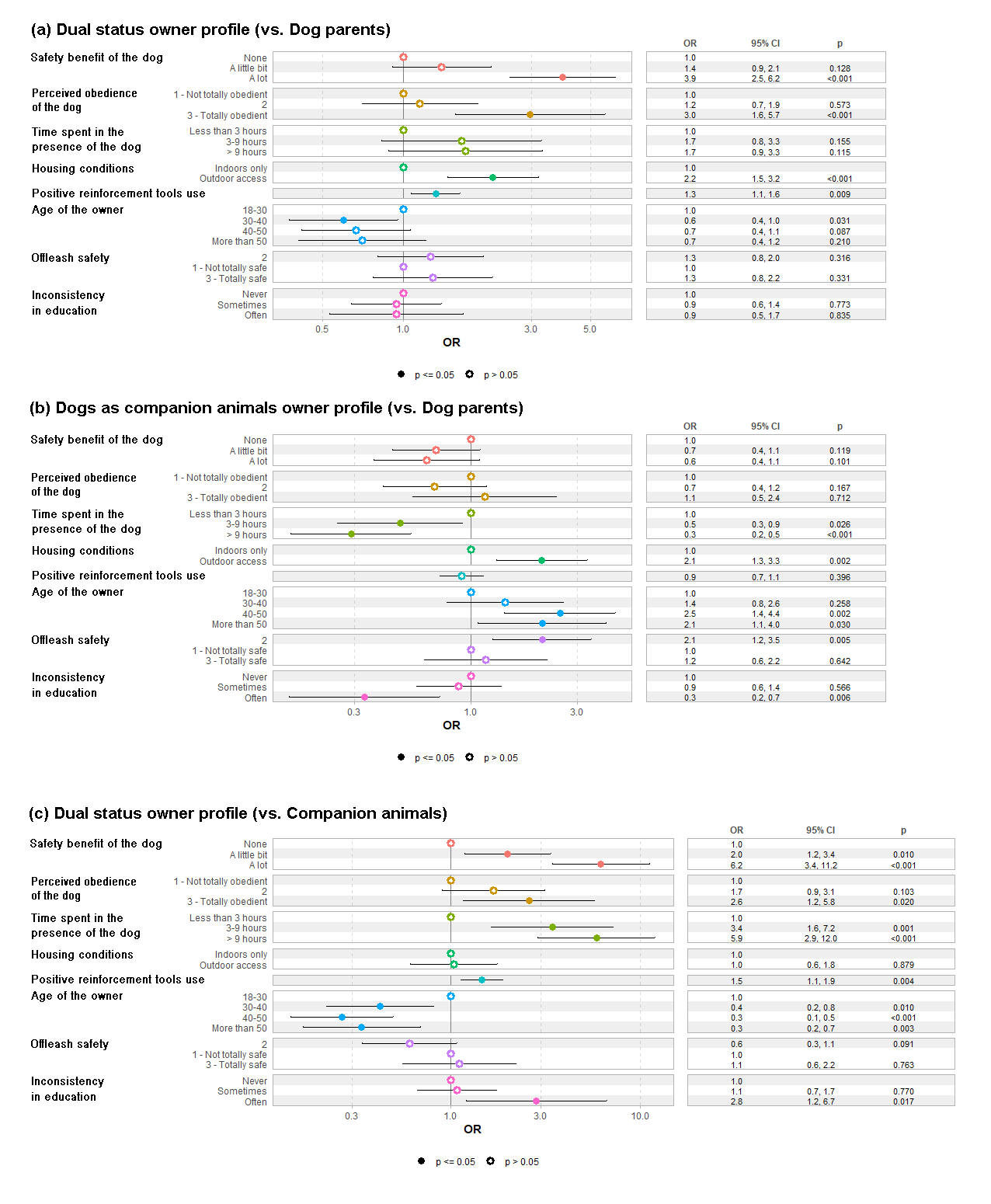
**

**Supplementary Figure S1**

Differences in multinomial logit coefficients across dog owner profiles. Reference group for comparisons across the predictor variables are indicated in brackets.
